# Spectroscopy of the Surface Polaritons in the Cd_X_Zn_(1−X)_P_2_ Solid Solutions

**DOI:** 10.1186/s11671-017-1880-8

**Published:** 2017-02-06

**Authors:** K. V. Shportko, T. R. Barlas, J. Baran, V. M. Trukhan, T. V. Shoukavaya, E. F. Venger

**Affiliations:** 1 Lashkaryov Institute of Semiconductor Physics, National Academy of Sciences of Ukraine, Nauki av. 45, Kyiv, 03028 Ukraine; 2 Institute of Low Temperature and Structure Research, PAS, 2 Oko´lna Street, P.O. Box 1410, 50-950 Wroclaw 2, Poland; 3 Scientific and Practical Center for Materials Science, National Academy of Sciences of Belarus, P. Brovki str. 19, 220072 Minsk, Belarus

**Keywords:** Nanocluster, Solid solution, Vibrational properties, Surface polaritons, ATR, 70.78.40.F, 70.63.20.K

## Abstract

Here we report on the analysis of the effect of the doping of CdP_2_ single crystals by ZnP_2_ nanoclusters on the dispersion of the surface polaritons (SP). The ATR spectroscopic technique has been applied to excite the SP in the Cd_X_Zn_(1−X)_P_2_ system. Analysis of the obtained spectra has shown that the doping of CdP_2_ single crystals by ZnP_2_ nanoclusters result in the position and the width of the dispersion branches of the SP. This effect is more pronounced in the low frequency dispersion branches. These SP branches are originated from phonons which correspond to the motion of the cation sublattice.

## Background

The recent interest to exploring the properties of zinc and cadmium diphosphides ZnP_2_ and CdP_2_ is caused by the possibility of employing them in various devices, such as, temperature detectors, deflectometers of laser beams, photoconducting cells, magnetic sensors, extenders, and stabilizers of laser radiation, photovoltaic applications [1, 2]. Vibrational properties of ZnP_2_ and CdP_2_ have been previously reported in [3–6] in the wide temperature range. The effect of the doping of CdP_2_ by ZnP_2_ nanoclusters on the vibrational properties of the resulting solid solutions of Cd_X_Zn_(1−X)_P_2_ have recently been presented in [7]. According to the technology of the obtaining Cd_X_Zn_(1−X)_P_2_ solid solutions, most of the ZnP_2_ nanoclusters are located in the near surface area. It has been shown [8], that surface polaritons are very sensitive to the presence of the surface defects and impurities. The dispersion and the damping of surface polaritons, that are localized in a thin surface layer with the thickness of the order of the reciprocal value of the damping constant, are very sensitive to the characteristics of the surface including the structure of the crystal and its relief [9]. It was shown that the optical spectroscopy is a powerful experimental technique to study the properties of complex structures [10, 11], and the most efficient way to obtain the data on the dispersion of SP in solid solutions is employing ATR technique, as it has been shown in [9] for Ga_1−x_Al_x_As and GaAs_x_P_1−x_. Thus, present work is aimed to study the influence of ZnP_2_ nanoclusters on the dispersion of the SP in Cd_X_Zn_(1−X)_P_2_ solid solutions. This might provide information on the distribution of the ZnP_2_ nanoclusters in the host CdP_2_ that can be useful for employing Cd_X_Zn_(1-X)_P_2_ in the construction of the optoelectronic devices.

The paper consists of the following parts: Introduction briefly represents the previous results and describes the motivation of the research. Experimental section procedures work, we describe the experimental procedures, such as preparing the samples, optical spectra measurements, and their treatment. In the Results and Discussion section we describe the influence of the doping CdP_2_ by the ZnP_2_ nanoclusters on the dispersion of SP in Cd_X_Zn_(1−X)_P_2_ by the analysis of the systematic changes in the ATR spectra. In Conclusions section we summarize obtained results.

## Methods

CdP_2_ in the polycrystalline form was grown from the initial elements by two-temperature way and then was used to grow single crystals of CdP_2_. The Cd_X_Zn_(1−X)_P_2_ solid solution was obtained in the following way: Zn was deposited on the surface of the CdP_2_ single crystal and then annealed in the oven at the temperature of 650 °C for 600 h. The Cd_X_Zn_(1−X)_P_2_ system is a CdP_2_ single crystal with inclusions of tetragonal ZnP_2_ with size of up to 100 nm [1]. The concentration of ZnP_2_ nanoclusters has been controlled by XRF, and in the studied Cd_X_Zn_(1−X)_P_2_ sample it was *x* = 0.9991_._ As a reference, in this work we also used pure CdP_2_ (*x* = 1) samples. All studied samples were in the shape of plates with a size of 2 × 3 × 1 mm.

The ATR spectra of the SP were recorded in the usual manner in the 150 − 500 cm^−1^ frequency range. In the experiments, we used p-polarized radiation and spectrometers KSDI-82 equipped with ATR unit LOMO NPVO-1 as well as Bruker IFS 88 equipped with Perkin Elmer ATR unit. CsI semicylinder served as ATR element in both cases. ATR spectra were recorded with several angles of the incidence of the radiation in the range of 40–60°. The polystyrene spacers were used to make an air gap between the investigated sample and the semicylinder, it was varied from 6 to 8 μ.

## Results and Discussion

**Fig. 1:**
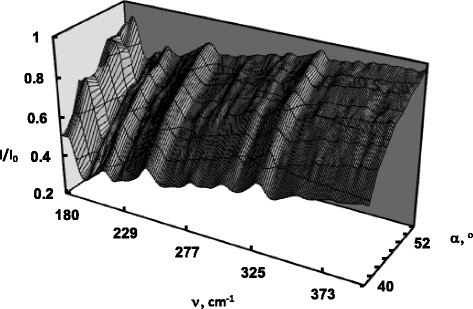
Experimental ATR surface *I*(*α,ν*)*/I*_*0*_(*α,ν*) of CdP_2_ (*x* = 1)

**Fig. 2:**
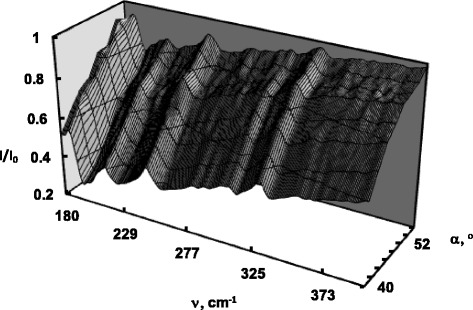
Experimental ATR surface *I*(*α,ν*)*/I*_*0*_(*α,ν*) of Cd_X_Zn_(1−X)_P_2_ (*x* = 0.9991)

**Fig. 3:**
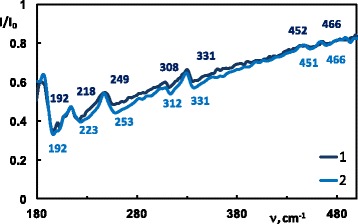
Experimental ATR spectra of Cd_X_Zn_(1−X)_P_2_ recorded at 42°: 1 (*dark blue*) *x* = 1, 2 (*light blue*) *x* = 0.9991

To study the dispersion of SP, the method of ATR was employed. The general principles of the excitation of surface waves by ATR has been proposed and described by Otto in [12] for the surface plasma waves on metals. Figures 1 and 2 represent experimental ATR spectra of CdP_2_ and Cd_X_Zn_(1−X)_P_2_ in form of surfaces *I*(*α,ν*)*/I*_*0*_(*α,ν*), which is a three-dimensional presentation of the system’s response that depends on the radiation frequency *ν* and angle of its incidence *α*. Each surface is formed by eight experimental ATR spectra obtained in the range of 42–55° and in the presence of the SP damping and dissipation of the electromagnetic wave energy the surfaces *I*(*α,ν*)*/I*_*0*_(*α,ν*) exhibit 7 “canyons” connected with several “passes”. The depth of the “canyon” depends on the gap *d* between the ATR semicylinder and sample, radiation frequency *ν*, dielectric permittivity *ε*(*ν*) of the sample, refractive indexes of the ATR unit and gap. The SP dispersion curves *ν*_*s*_(*k*) correspond to the “canyons”, i.e., to the set of ATR spectra minima. In order to better show the impact of the doping, we plotted the experimental ATR spectra of Cd_X_Zn_(1−X)_P_2_ (*x* = 1 and *x* = 0.9991) recorded at 42° in Fig. 3.

**Fig. 4:**
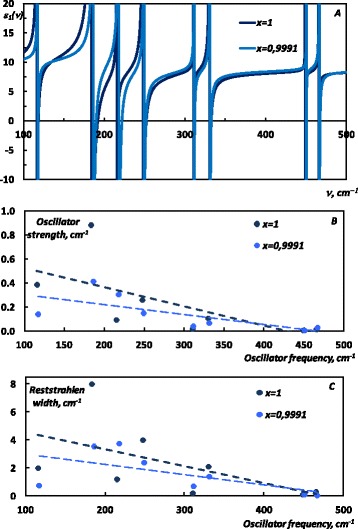
Impact of the doping on the reststrahlen bands in Cd_X_Zn_(1−X)_. The dielectric function of of Cd_X_Zn_(1−X)_. **a** The real part of the dielectric function. **b** Distribution of the oscillator strength (Lorentz oscillator model) vs. corresponding oscillator wavenumber. **c** Widths of the reststrahlen bands vs. corresponding oscillator wavenumber

The analysis of the obtained experimental data on SP in Cd_X_Zn_(1−X)_P_2_ we begin with the spectral ranges of their existence. SP propagate along the interface and decay exponentially for directions normal to the interface between two media, one having a negative dielectric permittivity and the other a positive one. Tetragonal β-CdP_2_ and α-ZnP_2_ belong to the similar space symmetry group *P*4_1_2_1_2 $\left({D}_4^4\right)$ [13], where the vibrational modes have following symmetry types: 9*A*_1_ + 9*A*_2_ + 9*B*_1_ + 9*B*_2_ + 18*E,* according to the results of the group theoretical analysis [14]*.* Modes of the symmetry *A*_2_(*z*) and *E*(*x,y*) modes are IR active, whereas *A*_1_, *B*_1_, *B*_2_, *E* are first-order Raman active. Infrared active *A*_2_(*z*) and *E*(*x,y*) modes in CdP_2_ and ZnP_2_ cause occurrence of the several reststrahlen bands in the corresponding dielectric function that have been reported in [3]. In [7], we analysed the effect of the doping of the CdP_2_ single crystal by the ZnP_2_ nanoclusters on the vibrational properties. Due to the similarity of the crystal structure of ZnP_2_ and CdP_2_, their dielectric functions exhibit similar profile in the IR, exhibiting the similar number and types of the modes. In Fig. 4a, we show the real part of the dielectric function *ε*_*1*_ (with zero phonon damping) of Cd_X_Zn_(1−X)_P_2_ and CdP_2_ obtained from the reflectance measurements [7]. Replacing Cd by Zn in Cd_X_Zn_(1−X)_P_2_ causes evolution of the reststrahlen bands located at lower wavenumbers, that is shown in the Fig. 4: these bands are shifted to the higher wavenumbers, and at the same time they have smaller widths in the comparison to those of the corresponding reststrahlen bands in pure CdP_2_. These findings can be explained in the terms of the electronic polarizability of the vibrating ions and their masses. In tetragonal CdP_2_, ZnP_2_ anion atoms form the zigzag chains which penetrate through the crystal [13]. In [15], the low frequency lattice vibrations have been attributed to the Zn(Cd)-P and Zn(Cd)-Zn(Cd) modes, whereas the high frequency peaks were assigned to the internal vibrations of the phosphorus chain. Therefore, in comparison with CdP_2_ in Cd_X_Zn_(1−X)_P_2_ the most changes should occur with the low frequency cation vibrations, whereas the high frequency vibrations of the phosphorus chain should remain mostly unchanged. Difference in the masses of Zn and Cd is responsible for the blue shift of the reststrahlen bands that correspond to the cation-cation and cation-anion modes, since different energies are needed for the excitement of the lite Zn and heavy Cd ions. Observed evolution of the widths of the bands can be explained in the terms of the electronic polarizability of the vibrating ions. According to [16], Cd ions exhibit significantly higher polarizability, and therefore, the replacement of the Cd ions by Zn ones, upon forming the ZnP_2_-nanoclasers, reduces the corresponding dipole moment, which we detected in the reduced oscillator strength and the shortening of the spectral width of the corresponding reststrahlen bands, that are presented in Table 1.

**Table 1:** The position and width of the reststrahlen bands in Cd_X_Zn_(1−X)_P_2_

No.	*x* = 1		*x* = 0.9991	
	*ν*_1_, cm^−1^	Δ*ν*, cm^−1^	*ν*_1_, cm^−1^	Δ*ν*, cm^−1^
1	183.3	8.00	189.65	3.55
2	215.0	1.20	221.55	3.75
3	247.6	4.00	251.5	2.40
4	310.4	0.20	312.0	0.70
5	330.2	2.10	332.5	1.40
6	448.9	0.10	450.95	0.10
7	465.7	0.30	467.05	0.05

**Fig. 5:**
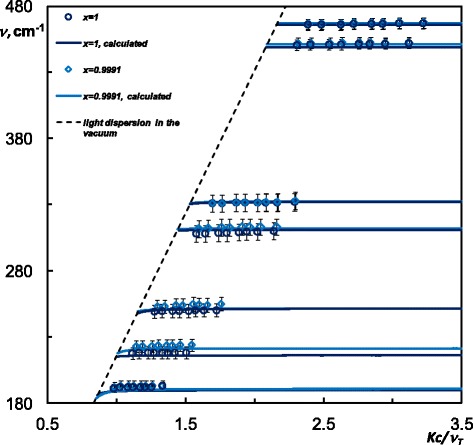
Experimental and calculated dispersion of the SP in Cd_X_Zn_(1−X)_: *x* = 1(pure CdP_2_) and *x* = 0.9991(solid solution)

The dispersion of the SP *ν*_*s*_ *(k)* in CdP_2_ and Cd_X_Zn_(1−X)_P_2_ has been calculated in the same manner as previously in [17, 18] with respect to the sample orientation (*C*||*y*) and using as the input data the discussed above dielectric function of CdP_2_ and Cd_X_Zn_(1−X)_P_2_ within the spectral range of the ATR measurements. According to the Table 1, in both CdP_2_ and Cd_X_Zn_(1−X)_P_2_ calculated dispersion curve *ν*_*s*_ *(k)* of the SP exhibits 7 branches within studied range. From the obtained ATR spectra, we have also distinguished 7 experimental dispersion curves to the SP in each CdP_2_ and Cd_X_Zn_(1−X)_P_2_ within the measured spectral range. However, the minima in the ATR spectra that correspond to the bands between 450 and 467 cm^−1^ were hardly distinguished from the noise due to the weakness of the corresponding modes: the last can also be noticed in the reflectance data [7]. The SP dispersion curves have been evaluated from the ATR spectrum minima in the following way:

1 $$ k=\left(2\pi \nu / c\right)\; n \sin \alpha, $$

here *ν* is the frequency of the ATR spectrum minimum; *c* is the speed of light in a vacuum; *n* is the refractive index of material of the ATR semicylinder (*n* = 1.72, CsI). Thus, we have shown that experimental SP dispersion, shown in dots in Fig. 5, obtained from the ATR spectra is in reasonable agreement with calculated branches.

## Conclusions

We applied the spectroscopy of the SP to study the effect of the doping of CdP_2_ by ZnP_2_ nanoclusters on the properties of the near surface area of Cd_X_Zn_(1−X)_P_2_ solid solution. Presence of ZnP_2_ nanoclusters in the near surface area of Cd_X_Zn_(1−X)_P_2_ causes shift of the reststrahlen bands as well as the shortening of their widths. This finding is confirmed by the observed evolution of the dispersion of the SP in Cd_X_Zn_(1−X)_P_2_. With obtained results, we have shown that the spectroscopy of the SP might be used as a non-destructive method of the property control of the near surface area of Cd_X_Zn_(1−X)_P_2_.
